# Prognostic parameters for recurrence of papillary thyroid microcarcinoma

**DOI:** 10.1186/1471-2407-8-296

**Published:** 2008-10-14

**Authors:** Tae Yong Kim, Suck Joon Hong, Jung Min Kim, Won Gu Kim, Gyungyub Gong, Jin Sook Ryu, Won Bae Kim, Sung-Cheol Yun, Young Kee Shong

**Affiliations:** 1 Department of Internal Medicine, Asan Medical Center, University of Ulsan College of Medicine, Seoul, Korea; 2 Surgery, Asan Medical Center, University of Ulsan College of Medicine, Seoul, Korea; 3 Pathology, Asan Medical Center, University of Ulsan College of Medicine, Seoul, Korea; 4 Nuclear Medicine, Asan Medical Center, University of Ulsan College of Medicine, Seoul, Korea; 5 Division of Biostatistics, Asan Medical Center, University of Ulsan College of Medicine, Seoul, Korea; 6 Research Institute and Hospital, National Cancer Center, Goyang, Gyeonggi, Korea

## Abstract

**Background:**

Papillary thyroid microcarcinoma (PTMC) is defined as a papillary thyroid carcinoma less than or equal to 1.0 cm in size. Independent prognostic factors for clinical recurrence of PTMC have not been clearly delineated.

**Methods:**

Clinicopathological parameters predicting PTMC recurrence were determined by retrospective analysis of 307 patients.

**Results:**

Of the 293 patients eligible for analysis, 14 (5%) had recurrence during a median follow-up time of 65 months. Recurrence was observed in 8 of 166 patients (0.5%) treated with total or near-total thyroidectomy; gender (P = 0.02) and presence of lateral cervical node metastases at initial surgery (P = 0.01) were associated with recurrence. Six of the 127 patients (0.5%) treated with hemi- or subtotal thyroidectomy experience recurrences, but no significant prognostic factor for recurrence was identified. Multivariate Cox-regression analysis showed that gender and cervical lymph node metastasis were significant variables

**Conclusion:**

PTMC showed very diverse disease extent and could not be regarded as indolent, relatively benign disease based on the primary tumor size. The extent of surgery should be based on prognostic parameters, such as gender and lateral neck node metastasis, in patients with PTMC.

## Background

Papillary thyroid microcarcinoma (PTMC) is defined as a papillary thyroid carcinoma equal to or less than 1 cm in size, including extrathyroid extensions and/or nodal metastases. Recent reports have suggested that the increasing incidence of thyroid cancer is predominantly because of the increased detection of small papillary cancers[1,2]. Most PTMCs are incidentally detected by histological analysis of thyroid glands removed for preoperatively diagnosed benign diseases, or are indirectly diagnosed because of the presence of enlarged metastatic cervical lymph nodes or distant metastases [3,4]. In Korea and Japan, however, where ultrasonography is frequently used in the initial examination of thyroid glands [5-7], the incidence of PTMC has increased explosively [8].

Although PTMC used to be considered a rather benign entity with a very long indolent course, recent findings indicate that PTMCs are frequently detected in patients displaying factors associated with poor prognosis, including multifocality, bilaterality, extrathyroid extension, and lymph node metastasis [3,4,9-15]. The factors independently predictive of clinical recurrence in patients with PTMC, however, have not been clearly determined [3,4,9-15]. Thus, optimal management of PTMC patients can range from observation without surgery at one extreme to total thyroidectomy with therapeutic neck dissection followed by radioiodine treatment at the other extreme, depending on the presence of aggressive characteristics [16]. To more clearly determine optimal management regimens for PTMC patients, it is important to determine factors prognostic for clinical recurrence. We therefore assessed prognostic parameters in 307 patients with PTMC.

## Methods

### Definitions

PTMC was defined as "incidentally detected" when found during the histological analysis of thyroid glands removed for preoperatively diagnosed benign disease, when detected by imaging methods during screening for other purposes, or when found in the course of screening for thyroid disease [16]. PTMC was defined as "occult" when it was undetectable at clinical examination and was indirectly diagnosed because of the presence of enlarged metastatic cervical lymph nodes or distant metastases [16].

### Patients

Between January 1996 and December 2002, 1,262 patients with papillary thyroid carcinoma underwent initial thyroid surgery performed by one surgeon (S.J.H.) at the Asan Medical Center, Seoul, Korea. Of these, 307 (24%) patients (32 men and 275 women), of mean age 46 ± 10 years (range, 23 to 70 yrs), had tumors ≤ 1.0 cm in diameter, including 292 patients with tumors of conventional type, 14 with follicular variants, and 1 with an oncocytic variant. The prevalence of PTMC increased over time, with the proportion of PTMCs amongst all papillary thyroid carcinomas being 17% (105 of 604 patients) in 1996–1999, 23% (106 of 416 patients) in 2000–2001, and 40% (96 of 242 patients) in 2002.

Of the 307 patients with PTMCs, 20 had occult PTMCs, with 19 diagnosed because of the presence of palpable metastatic cervical lymph nodes and 1 diagnosed when a distant lung metastasis was found. The remaining 287 patients had incidentally detected PTMCs; 271 PTMCs were detected during imaging for other purposes in screening for thyroid disease, and 16 were found during pathological examination of thyroid specimens obtained in the course of surgical resection for other conditions. Of these, 10 patients underwent procedures for cosmetic removal of large nodules, 4 were operated upon to remove toxic multinodular goiters, and 2 patients underwent surgery for Graves' disease.

Medical records of all subjects were retrospectively reviewed, and patient demographics (age and gender), histopathological findings (tumor size, multifocality, bilaterality, extrathyroid extension, and cervical lymph node metastasis), and clinical outcomes were analyzed. Central cervical lymph nodes were defined as lymph nodes at level VI (i.e., pretracheal, paratracheal, and prelaryngeal/Delphian lymph nodes), and patients with metastases in these lymph nodes were classified as N1a according to the 2002 TNM staging. Lateral cervical lymph nodes were defined to include all unilateral cervical lymph nodes except for those at level VI, as well as bilateral, contralateral cervical, and superior mediastinal lymph nodes; patients with metastases in these lymph nodes were classified as N1b according to the 2002 TNM staging.

Informed consent was obtained from all subjects at the time of surgery, and our institutional ethics committee approved this retrospective review protocol.

### Initial treatment

Hemi- or subtotal thyroidectomy was initially performed on 141 patients. These included 112 cases with preoperative cytological diagnoses of PTMC but with no evidence of extrathyroid extensions or lymph node metastases, and clear contralateral lobes on preoperative thyroid ultrasonography. A further 21 patients showed indeterminate preoperative cytology, including follicular neoplasms and suspicion of PTC (papillary thyroid carcinoma). The final 8 patients with PTMC were diagnosed when their thyroids were removed for preoperatively diagnosed benign diseases. Seven patients underwent further completion thyroidectomy. Thus, 134 (44%) patients underwent hemi- or subtotal thyroidectomy. Central neck node dissection was performed on 107 patients and lymph node metastases to the central neck node(s) were pathologically confirmed in 47 (stage N1a).

Total or near-total thyroidectomy was performed on 173 patients (56%), including 7 with completion thyroidectomy. Seventeen patients did not undergo neck dissection; these patients either chose total thyroidectomy following an inconclusive preoperative diagnosis to avoid a possible second surgery after unilateral operation, or they had disease conditions requiring total thyroidectomy because of bilateral underlying benign pathology. Routine central neck node dissection without modified radical neck dissection was performed on 129 patients, regardless of nodal metastasis observed during surgery. Central neck node dissection with modified radical neck dissection was performed on 27 patients; 20 with occult PTMC detected by cytologically confirmed enlarged cervical lymph nodes and 7 with suspected but not cytologically confirmed lateral neck nodes detected by ultrasonography. Central neck node metastasis with or without lateral neck node metastasis was confirmed in 24 pN1b and 68 pN1a patients.

For pathologic examination, the whole thyroid gland was cut into 5 mm slices, fixed, and examined to define the dimensions of malignant lesions and to note multifocality. Pathology slides were reviewed by one experienced pathologist (G.G).

Of the 173 patients who underwent total or near-total thyroidectomy, 163 were treated post-surgically with radioiodine (2.78–5.55 GBq), whereas 10 were not treated in this manner. Of the latter patients, none had an extrathyroid extension or lymph node metastasis by postoperative pathology and all were younger than 45 years. All patients received suppressive doses of L-T4. No patient received external beam radiation treatment (EBRT) as initial therapy.

### Subsequent follow-up and localization of persistent or recurrent lesions

Post-surgical physical examinations were performed every 3–6 months. The 163 patients who received radioiodine remnant ablation treatment underwent diagnostic WBS (whole body scan) with 148 MBq [I^131^], and measurements of serum thyroglobulin (Tg) levels during thyroid hormone withdrawal (THW) were made every 1–2 years. Patients with serum Tg>2 μg/liter during THW who were negative for thyroglobulin antibody (TgAb), patients positive for serum TgAb, and patients with clinical suspicion of recurrence, were examined using one or more non-radioiodine imaging methods, including neck ultrasonography, [F^18^]-deoxyglucose positron emission tomography (FDG-PET), and/or chest computed tomography, to localize normal and/or malignant thyroid tissues.

The remaining 144 patients (i.e., the 10 patients who underwent total or near-total thyroidectomy without remnant ablation and the 134 patients who underwent hemi- or subtotal thyroidectomy) received neck ultrasonography and serum thyroid-stimulating hormone (TSH) measurements annually. The FDG-PET, chest CT, and other imaging methods were employed when indicated.

### Definition of clinical outcome

"Recurrence" was defined as persistence and/or reappearance of disease after initial operation, as determined cytologically or histopathologically. "Regional recurrence" was defined as recurrence in nodes or the operative bed, and "local recurrence" was defined as recurrence in the remaining thyroid after initial hemi- or subtotal thyroidectomy. The time to recurrence was defined as duration between the date of first operation and date of detection of recurrence by diagnostic procedure (for those who recur) or last show-up date to our hospital (for those who do not recur).

### Statistical analysis

Categorical variables are presented as numbers and percentages, and were compared using chi-square or Fisher's exact test. Continuous variables are presented as mean ± SD and range.

The Kaplan-Meier method including the log rank test was used to compare recurrence. Univariate analyses were performed separately for each of the variables using Cox-regression. Variables for which the P value was < .2 in univariate analysis were included in a multivariate Cox-regression model. A backward elimination process was used to develop the final multivariate model, and adjusted hazard ratio (HR) and 95% confidence intervals (CI) were calculated. P value of < .05 were considered significant. All statistical analyses were performed using SAS release 9.1.

## Results

### Clinical parameters

The mean tumor size was 0.8 ± 0.2 cm (range, 0.1–1.0 cm). Of the 307 patients, 84% had tumors > 0.5 cm in size, 32% had multifocal tumors, 19% had bilateral tumors, 38% had extrathyroid extensions of tumors, 37% had central neck node metastases (pN1a), and 9% had lateral neck node metastases (pN1b). Distant metastasis at the time of presentation was observed in only one patient. Of the 174 patients with extrathyroid extensions, only one showed gross invasion involving adjacent neck structures (pT4a), whereas 173 showed microscopic minimal extrathyroid extensions (pT3).

### Comparison of baseline clinical characteristics according to surgical extent (Table 1)

**Table 1:** Baseline clinical characteristics relative to surgical extent and tumor multifocality

Baseline characteristics	Surgical extent					Tumor multifocality					Overall	
			
	Total or near-total (n = 173)		Hemi- or subtotal (n = 134)		*P *value^a^	Multifocal (n = 98)		Solitary (n = 209)		*P *value^a^		
Age at operation (> 45 yrs)	95	(55)	54	(40)	0.01	53	(54)	96	(46)	0.22	149	(49)
Gender (male)	16	(9)	16	(12)	0.46	9	(9)	23	(11)	0.69	32	(10)
Tumor size												
≤ 0.5 cm	29	(17)	20	(15)	0.07	9	(9)	40	(19)	0.04	49	(16)
> 0.5 cm and ≤ 0.8 cm	63	(36)	66	(49)		40	(41)	89	(43)		129	(42)
> 0.8 cm	81	(47)	48	(36)		49	(50)	80	(38)		129	(42)
Multifocal tumor	84	(49)	14	(10)	< .001	N/A		N/A		N/A	98	(32)
Bilateral tumor	57	(33)	N/A			57	(69)	N/A		N/A	57	(19)
Extrathyroid extension	77	(45)	39	(29)	0.006	47	(48)	69	(33)	0.02	116	(38)
Lymph node metastasis												
pNx	17	(10)	27	(20)	< 0.001	13	(13)	31	(15)	0.03	44	(14)
pN0	64	(37)	60	(45)		30	(31)	94	(45)		124	(40)
pN1a^b^	68	(39)	47	(35)		43	(44)	72	(34)		115	(37)
pN1b^c^	24	(14)	0			12	(12)	12	(6)		24	(8)
Remnant ablation	163	(94)	N/A			81	(83)	82	(39)	< .001	163	(53)
Total or near-total thyroidectomy	N/A		N/A			84	(86)	89	(43)	< .001	173	(56)

^a ^by Fisher's exact test except for tumor size and lymph node metastasis; by the chi-squared test for tumor size and lymph node metastasis.

^b ^Metastasis to level VI lymph nodes (pretracheal, paratracheal, and prelaryngeal/Delphian), classified as N1a in the 2002 TNM staging.

^c ^Metastasis to unilateral, bilateral, or contralateral cervical superior mediastinal lymph nodes, classified as N1b in the 2002 TNM staging.

Percentages are given in parentheses; N/A, not applicable.

Age at diagnosis and the rates of multifocality, extrathyroid extension, and lymph node metastasis differed significantly between patients who underwent total or near-total thyroidectomy and those who underwent hemi- or sub-total thyroidectomy (Table 1).

### Comparison of baseline clinical characteristics according to multifocality (Table 1)

When we assessed baseline characteristics relative to post-surgically determined multifocality, we found that tumor size was significantly larger in patients with multifocal tumors than in those with solitary tumors, as was the frequency of extrathyroid extension and lymph node metastasis, especially in the lateral neck nodes. In addition, patients with multifocal tumors more frequently underwent total or near-total thyroidectomy than did those with solitary tumors (Table 1).

### Clinical characteristics of patients with recurrence

Clinical recurrence was evaluated in 306 patients who initially presented with PTMCs but without distant metastases; of these, 293 patients were eligible for analysis, with a median follow-up time of 65 months (range 5–139 months). These included 166 of the 172 patients who underwent total or near-total thyroidectomy and 127 of the 134 who underwent hemi- or subtotal thyroidectomy. Of these 293 patients, 14 (5%) had clinical recurrences during follow-up (Table 2). Regional recurrence in nodes or the thyroid bed was observed in 9 patients (patients 1–9 in Table 2), and local recurrence in the thyroid was observed in 5 patients (patients 10–14 in Table 2). Of the 8 patients who underwent total or near-total thyroidectomy and had recurrences (patients 1 to 8 in Table 2), 7 had regional recurrences in cervical lymph nodes and 1 in the thyroid bed. Two of these patients showed newly developed pulmonary metastases (patients 5 and 7 in Table 2), which were detected by increases in serum Tg levels and FDG uptake on scanning, and the appearance of visible nodules on chest CT scans. These two patients, initially staged as pT1N1b and pT3N1b, respectively, underwent additional surgery followed by radioiodine treatment, but no iodide uptake was detected during post-treatment whole body scans. One patient (patient 5 in table 2) showed progressive multiple unresectable cervical, mediastinal, and lung metastases, and EBRT was performed to control locally advanced disease. The other case (patient 7 in Table 2) was treated by thyroxine suppression, and definitive evidence of disease progression was not observed by serial chest CT scan.

**Table 2:** Clinical characteristics of patients with clinical recurrence

Patient No.	1	2	3	4	5	6	7
Age/Sex	46/M	32/M	43/M	38/F	60/F	44/F	63/F
Initial Surgical extent	TT, MRND	TT, CND	TT, MRND	TT, MRND	TT, MRND	TT, CND	TT, MRND
I-131 Remnant ablation	done	Done	done	done	Done	done	done
Just before recurrence							
sTg (ng/mL)	58.6	34.4	17.3	13.8	33	21.5	20.7
TgAb	negative	Negative	negative	positive	negative	negative	negative
Disease free survival (months)	9	11	13	24	31	35	43
Type of recurrence (Level of recurrent node confirmed by reoperation)^c^	regional (i/l II, III, IV)	regional (i/l III, SCN)	regional (i/l II, III, IV)	regional (i/l II, III, IV, SCN, VII)	regional (thyroid bed)	regional (i/l IV; c/l III, IV)	regional (i/l II, III, IV)
Additional treatment	surgery, ^131^I	surgery, ^131^I	surgery, ^131^I, EBRT	surgery	surgery, ^131^I, EBRT	surgery, ^131^I	surgery, ^131^I
Follow-up							
sTg (ng/mL)	UD	12.6	1.1	8.7	944^a^	12.4	40.0^b^
TgAb	negative	negative	negative	positive	negative	negative	negative

Patient No.	8	9	10	11	12	13	14

Age/Sex	42/F	50/F	40/F	43/F	32/F	40 M	33/M
Initial Surgical extent	TT, MRND	STT	LOB	LOB	LOB, CND	LOB, CND	LOB, CND
I-131 Remnant ablation	done	not done	not done	not done	not done	not done	not done
Just before recurrence							
sTg (ng/mL)	1.1	N/A	N/A	N/A	N/A	N/A	N/A
TgAb	neg	N/A	N/A	N/A	N/A	N/A	N/A
Disease free survival (months)	99	8	10	15	44	53	63
Type of recurrence (Level of recurrent node confirmed by reoperation)^c^	regional (i/l II, III)	regional (i/l III, IV)	local (c/l lobe)	local (c/l lobe)	local (c/l lobe)	local (c/l lobe)	local (c/l lobe)
Additional treatment	surgery, ^131^I	surgery, ^131^I	follow-up loss	surgery, ^131^I	surgery, ^131^I	surgery, ^131^I	surgery, ^131^I
Follow-up							
sTg (ng/mL)	UD	UD		UD	UD	UD	UD
TgAb	negative	positive		negative	negative	negative	negative

^a ^Chest CT suggested multiple lung metastases. This patient underwent additional surgery and radioiodine treatment, but no iodide uptake was noted during a post-treatment whole body scan. This patient, however, showed progressive, multiple, unresectable, cervical, mediastinal, and lung metastases, and EBRT was performed to control locally advanced disease.

^b ^Chest CT and FDG-PET suggested multiple lung metastases. This patient underwent additional surgery and radioiodine treatment, but no iodide uptake was noted during a post-treatment whole body scan. This was followed by thyroxine suppression, with no definitive evidence of disease progression observed on serial chest CT scans.

sTg, stimulated thyroglobulin; TT, total thyroidectomy; STT, subtotal thyroidectomy; LOB, lobectomy; CND, central neck node dissection; MRND, modified radical neck dissection; UD, undetectable; N/A, not applicable; i/l, ipsilateral; c/l, contralateral; EBRT, external beam radiation treatment.

Of the 6 patients who underwent hemi- or subtotal thyroidectomy (patients 9 to 14 in Table 2), 1 suffered a regional recurrence (patient 9) and 5 had local recurrences in the contralateral lobe of the remaining thyroid (patients 10–14 in Table 2). Five patients (patients 9 and 11 to 14 in Table 2), who were treated by re-operation followed by radioiodine therapy, achieved complete negative conversion of stimulated serum Tg level.

### Univariate analyses of factors predicting clinical recurrence according to surgical extent (Table 3)

**Table 3:** Univariate analyses of clinical recurrence with clinicopathological parameters in 293 patients with PTMC, according to surgical extent

Baseline characteristics	Patients with total or near-total thyroidectomy (n = 166)			Patients with hemi- or subtotal thyroidectomy (n = 127)			Overall (n = 293)		
			
	HR	95% CI	*P*	HR	95% CI	*P*^a^	HR	95% CI	*P*
Age at operation									
≤ 45 yrs	1.88	0.45–7.90	0.39	3.19	0.37–27.3	0.29	2.28	0.71–7.27	0.16
> 45 yrs	1.00	-	-	1.00	-	-	1.00	-	-
Gender									
Men	5.92	1.41–24.8	0.02	4.6	0.83–25.4	0.96	5.22	1.74–15.6	0.003
Women	1.00	-	-	1.00	-	-	1.00	-	-
Tumor size									
Per 1 mm increase	1.21	0.82–1.78	0.35	1.01	.0.68–1.51	0.96	1.11	0.85–1.45	0.45
Multifocality									
Solitary tumor	1.48	0.35–6.19	0.59	0.25	0.05–1.36	0.11	0.87	0.29–2.59	0.8
Multifocal tumor	1.00	-	-	1.00	-	-	1.00	-	-
Extrathyroid extension									
Absent	0.5	0.12–2.09	0.34	0.37	0.08–1.86	0.23	0.45	0.16–1.31	0.14
Present	1.00	-	-	1.00	-	-	1.00	-	-
Lymph node metastasis			0.08			0.83			0.049
pNx	0	NC	1	1.64	0.33–8.14	0.54	1.62	0.39–6.79	0.51
pN0	0	NC	1	0	NC	0.99	0	NC	0.99
pN1a^a^	1.00	-	-	1.00	-	-	1.00	-	-
pN1b^b^	8.25	1.66–40.9	0.01	N/A			5.25	1.60–17.3	0.51
Bilaterality									
Unilateral tumor	0.71	0.17–2.97	0.64	N/A			0.81	0.23–2.90	0.74
Bilateral tumor	1.00	-	-				1.00	-	-
Remnant ablation									
Not done	0	NC	1	N/A			N/A		
Done	1.00	-	-						

^a ^Metastases to level VI lymph nodes (pretracheal, paratracheal, and prelaryngeal/Delphian lymph nodes), classified as N1a in the 2002 TNM staging.

^b ^Metastases to unilateral, bilateral, or contralateral cervical superior mediastinal lymph nodes, classified as N1b in the 2002 TNM staging.

Percent recurrence rates were given in parentheses; HR, hazard ration; CI, confidence interval; N/A, not applicable; INF, infinite; NC, cannot calculate

**Figure 1:**
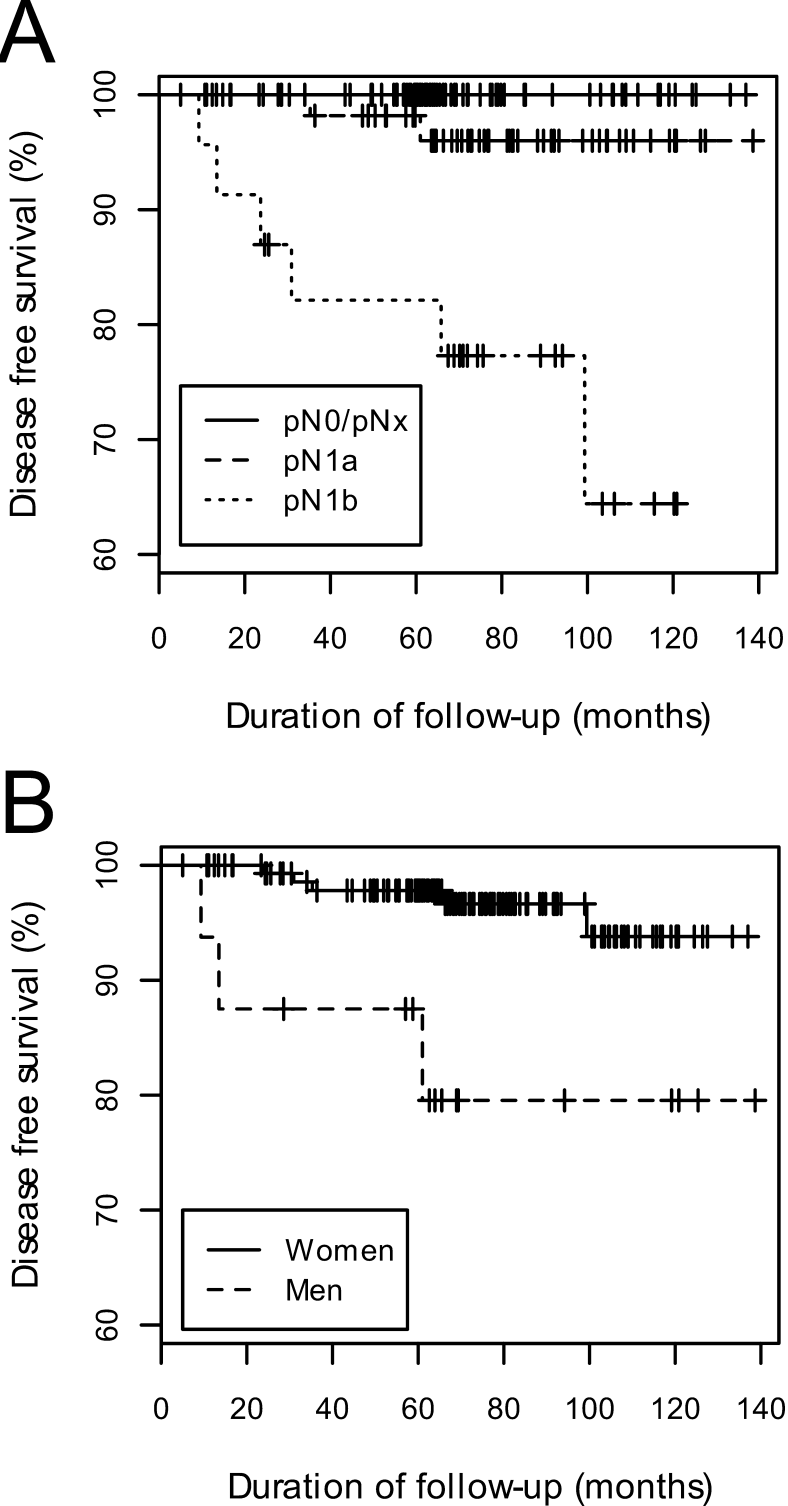
**Kaplan-Meier curves of disease-free survival according to (A) lymph node metastasis and (B) gender in 166 patients with papillary thyroid microcarcinoma who underwent total or near-total thyroidectomy**. The curves for pN0 and pNx overlapped because no recurrence was observed in either group.

**Figure 2:**
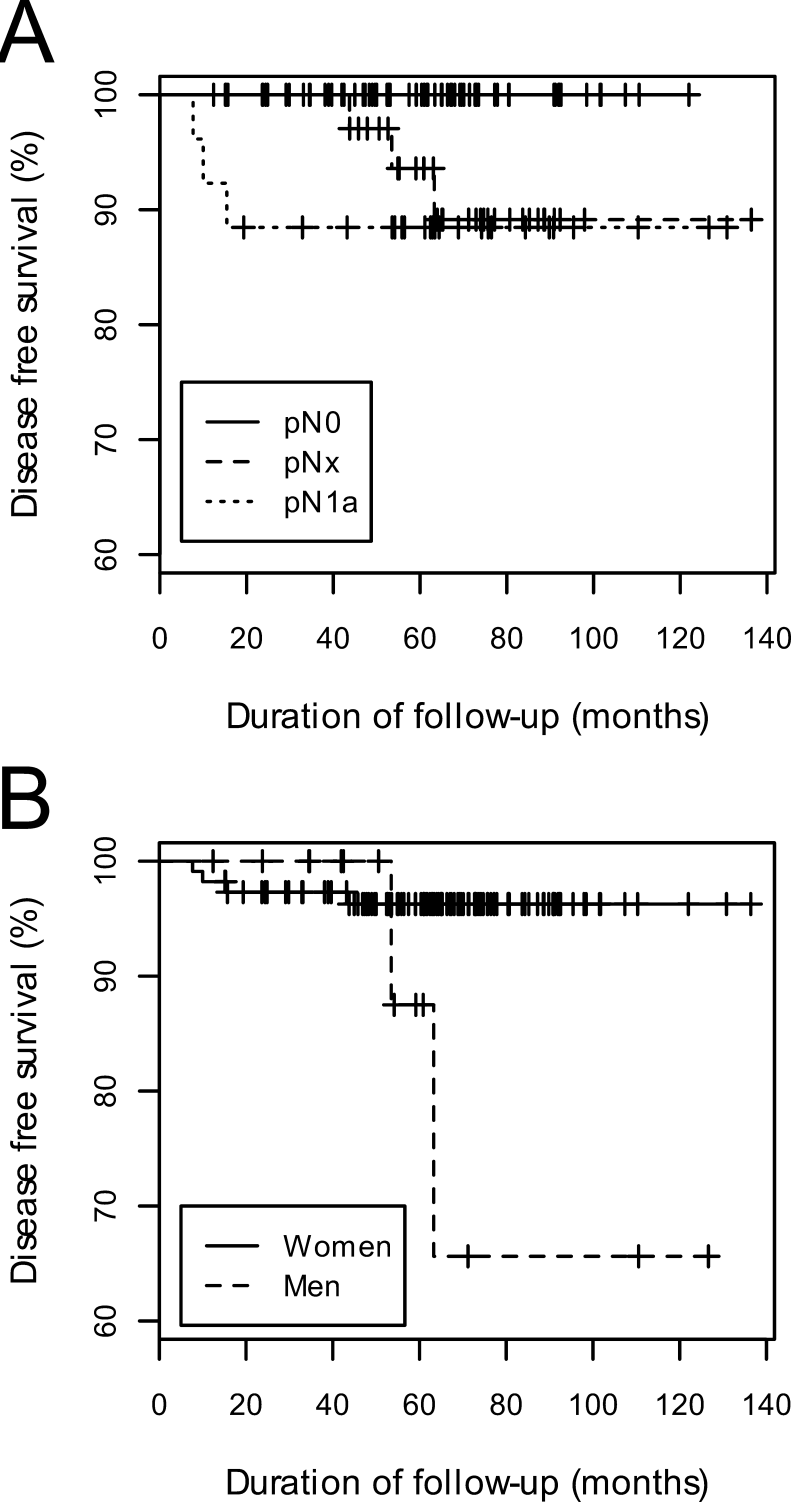
Kaplan-Meier curves of disease-free survival according to (A) lymph node metastasis and (B) gender in 127 patients with papillary thyroid microcarcinoma who underwent hemi- or subtotal thyroidectomy.

Univariate analysis showed that, in patients who underwent total or near-total thyroidectomy, presence of cervical lymph node metastasis at initial surgery (p = 0.01, Fig. 1A) and gender (p = 0.02, Fig 1B) were significantly associated with clinical recurrence. In patients who underwent partial thyroidectomy, however, there were no significant associations between clinicopathological parameters and clinical recurrence. The Kaplan-Meier estimate curves for cervical lymph node metastasis and gender are shown in Fig. 2A and Fig. 2B, respectively. Amongst the overall patient cohort, regardless of surgical extent, the presence of cervical neck lymph node metastases at initial surgery (p = 0.049) and gender (p = 0.003) were significantly associated with tumor recurrence.

### Subgroup analyses of factors predicting clinical recurrence according to surgical extent after excluding patients with pNx staging (Table 4)

**Table 4:** Subgroup analyses of association of clinical recurrence in 251 patients with PTMC according to surgical extent after excluding patients without pathologic N staging information

Baseline characteristics	Patients with total or near-total thyroidectomy (n = 150)			Patients with hemi- or subtotal thyroidectomy (n = 101)			Overall (n = 251)		
			
	HR	95% CI	*P*	HR	95% CI	*P*	HR	95% CI	*P*
Age at operation									
≤ 45 yrs	1.75	0.41–7.39	0.45	INF	NC	0.99	2.38	0.63–8.98	0.20
> 45 yrs	1.00	-	-	1.00	-	-	1.00	-	-
Gender									
Men	5.35	1.28–22.4	0.02	24.2	2.17–270	0.01	7.59	2.31–25.0	0.001
Women	1.00	-	-	1.00	-	-	1.00	-	-
Tumor size									
Per 1 mm increase	1.17	0.79–1.72	0.44	1.36	.0.67–2.76	0.4	1.23	0.86–1.74	0.26
Multifocality									
Solitary tumor	1.58	0.38–6.62	0.53	0.18	0.02–1.93	0.16	0.9	0.26–3.08	0.86
Multifocal tumor	1.00	-	-	1.00	-	-	1.00	-	-
Extrathyroid extension									
Absent	0.53	0.13–2.23	0.39	INF	NC	1	0.77	0.23–2.52	0.66
Present	1.00	-	-	1.00	-	-	1.00	-	-
Lymph node metastasis			0.04			1			0.03
pN0	0	NC	1	0	NC	0.99	0	NC	0.99
pN1a^a^	1.00	-	-	1.00	-	-	1.00	-	-
pN1b^b^	8.25	1.66–40.9	0.01	N/A			5.14	1.56–17.0	0.007
Bilaterality									
Unilateral tumor	0.74	0.18–3.11	0.69	N/A			0.65	0.17–2.47	0.53
Bilateral tumor	1.00	-	-				1.00	-	-
Remnant ablation									
Not done	0	NC	1	N/A			N/A		
Done	1.00	-	-						

^a ^Metastases to level VI lymph nodes (pretracheal, paratracheal, and prelaryngeal/Delphian lymph nodes), classified as N1a in the 2002 TNM staging.

^b ^Metastases to unilateral, bilateral, or contralateral cervical superior mediastinal lymph nodes, classified as N1b in the 2002 TNM staging.

Percent recurrence rates were given in parentheses; CI, confidence interval; N/A, not applicable; INF, infinite; NC, cannot calculate; HR, hazard ratio.

Subgroup analysis was also performed on the 251 PTMC patients on whom information on pathologic N staging was available, after excluding the 42 patients with pNx staging. Amongst patients who underwent total or near-total thyroidectomy, gender (p = 0.02) and presence of cervical lymph node metastasis at initial surgery (p = 0.04) were significantly associated with clinical recurrence on univariate analysis. Gender (p = 0.01) was also significantly associated with clinical recurrence amongst patients who underwent hemi- or subtotal thyroidectomy. Amongst the overall patient cohort, regardless of surgical extent, gender (p = 0.03) and the presence of neck lymph node metastasis at initial surgery (p = 0.03) were significantly associated with tumor recurrence.

### Multivariate Analyses of predicting factors for clinical recurrence according to surgical extent (Table 5 and 6)

**Table 5:** Multivariate analyses of clinical recurrence with clinicopathological parameters in 293 patients with PTMC according to surgical extent

Baseline characteristics	Patients with total or near-total thyroidectomy (n = 166)			Patients with hemi- or subtotal thyroidectomy (n = 127)			Overall (n = 293)		
			
	HR	95% CI	*P*	HR	95% CI	*P*	HR	95% CI	*P*
Gender									
Men	4.58	1.08–19.5	0.04	4.6	0.83–25.4	0.08	4.9	1.62–14.8	0.005
Women	1.00	-	-	1.00	-	-	1.00	-	-
Lymph node metastasis			0.12						0.049
pNx	0	NC	0.99				1.85	0.44–7.79	0.4
pN0	0	NC	1				0	NC	0.99
pN1a^a^	1.00	-	-				1.00	-	-
pN1b^b^	7.32	1.46–36.7	0.015				4.91	1.49–16.2	0.009

^a ^Metastases to level VI lymph nodes (pretracheal, paratracheal, and prelaryngeal/Delphian lymph nodes), classified as N1a in the 2002 TNM staging.

^b ^Metastases to unilateral, bilateral, or contralateral cervical superior mediastinal lymph nodes, classified as N1b in the 2002 TNM staging.

HR, hazard ratio; CI, confidence interval; NC, cannot calculate

**Table 6:** Multivariate subgroup analyses of association of clinical recurrence in 251 patients with PTMC according to surgical extent after excluding patients without pathologic N staging information

Baseline characteristics	Patients with total or near-total thyroidectomy (n = 150)			Patients with hemi- or subtotal thyroidectomy (n = 101)			Overall (n = 251)		
			
	HR	95% CI	*P*	HR	95% CI	*P*	HR	95% CI	*P*
Gender									
Men	4.58	1.08–19.5	0.04	24.2	2.17–270	0.01	6.51	1.97–21.5	0.002
Women	1.00	-	-	1.00	-	-	1.00	-	-
Lymph node metastasis			0.053						0.04
pN0	0	NC	0.99				0	NC	0.99
pN1a^a^	1.00	-	-				1.00	-	-
pN1b^b^	7.32	1.46–36.7	0.015				4.71	1.42–15.6	0.01

^a ^Metastases to level VI lymph nodes (pretracheal, paratracheal, and prelaryngeal/Delphian lymph nodes), classified as N1a in the 2002 TNM staging.

^b ^Metastases to unilateral, bilateral, or contralateral cervical superior mediastinal lymph nodes, classified as N1b in the 2002 TNM staging.

HR, hazard ratio; CI, confidence interval; NC, cannot calculate

Multivariate analysis, both for all 293 eligible PTMC patients (table 5) and for the 251 patients remaining after exclusion of those with pNx staging (table 6), showed that cervical node metastasis and gender were independently associated with clinical recurrence

## Discussion

Most PTMCs are incidentally detected during the histological analysis of thyroid glands removed for preoperatively diagnosed benign disease, or are indirectly diagnosed because of the presence of enlarged metastatic cervical lymph nodes or distant metastases [3,4]. A smaller proportion of these tumors is detected during imaging of the neck for other purposes, such as parathyroid evaluation, using (for example) carotid Doppler, CT, and FDG-PET. In Korea and Japan, however, many health care centers perform ultrasonography during initial examination of the thyroid [5-7], and impalpable nodules have increasingly been discovered. Korea is a country without iodine deficiency [17] and many thyroids have only one nodule, making ultrasound an excellent method to detect PTMCs. Furthermore, the incidence of PTMC diagnosed by ultrasonography screening for thyroid disease, and by fine needle aspiration cytology (FNAC) guided by ultrasonography, has increased dramatically, as has the proportion of PTMC procedures amongst all thyroid cancer surgeries, to attain a level of about 50% in Korea [8]. We found that the proportion of PTMCs amongst all papillary thyroid carcinomas increased over time, from 16% to 40% between 1996 and 2002, a finding in agreement with previous reports [1,2,18]. Only 5% of PTMCs were incidentally detected during pathological examinations of thyroid specimens resected for other diseases; this figure is lower than the 21% to 67% previously reported [3,4,10,15]. This may be because of the increased incidence of PTMC diagnosed by screening ultrasonography and FNAC in Korea.

We found that recurrence in some patients was detected after a very short follow-up period. These patients were evaluated before and after surgery by ultrasonography, probably the most useful method for discovery of PTMC node metastases, with a positive predictive value greater than 80% [19]. However, the sensitivity and negative predictive value of ultrasonography for diagnosis of node metastasis were 38% and 66%, respectively [12]. Furthermore, PTMC patients without evidence of positive lymph nodes by ultrasonography did not undergo neck dissection, so their exact nodal staging remains unclear. Ultrasonography has been reported to be relatively insensitive (~50%) for discovery of small PTMC foci in the contralateral lobe [12], suggesting that many false-negative findings may be made even when ultrasonography is performed by skilled operators. Thus, the relatively short period to recurrence after initial surgery may be because of limitations caused by the relatively low sensitivity of preoperative ultrasonographic evaluation. Nevertheless, this method should be regarded as the single most important tool to determine the extent of surgery.

Cervical lymph node metastasis has been reported to occur in 12–64% of PTMC patients [3,4,9-15] and may be a PTMC prognostic factor [9-15]. Recently, however, pathologically confirmed lateral neck metastasis, not central neck node metastasis, was reported to be a strong predictor of relapse in PTMC patients [12,13]. Our multivariate analysis also found that lateral cervical node metastasis was the most powerful independent predictor of clinical recurrence, after adjusting for other variables.

Although we found that the male gender was significantly associated with a higher clinical recurrence rate, the association was lost after adjusting for other variables. In contrast, no previous studies have reported an association of gender with clinical recurrence in PTMC patients [3,4,9-15], suggesting that additional studies are needed to confirm this association.

We also found that microscopic extrathyroid extension was not associated with poor prognosis in PTMC patients, a finding in agreement with previous results [3,4,10-15]. Further studies are needed to clarify whether the presence of a pT3 lesion is an independent risk factor for poor prognosis. Previous studies have reported that 42–79% of PTMC patients had tumors ≥ 0.5 cm in size [3,4,9-11,14,15], and 15–40% had multifocal tumors [3,4,9-11,14,15]. Tumor recurrence may be associated with tumor size [4] and multifocality [11,15]. We also observed an association between multifocality and tumor size, as well as with greater extrathyroid extension and more extensive cervical nodal recurrence. In comparison with previous studies, which reported that 70–81% of patients with recurrence had multifocal tumors at initial presentation, we found that only 36% of clinical recurrences were associated with multifocal disease in this study, and that this multifocality was not significantly associated with clinical recurrence. We cannot explain this discrepancy, but initial total or near-total thyroidectomy followed by the use of radioactive iodine ablation in our patients may have obviated tumor recurrence in some patients with multifocal disease.

Although previous studies reported that surgical extent may influence clinical outcome [3,14], we found that recurrence rates were similar in patients who underwent hemi- or subtotal thyroidectomy to those in patients receiving total or near-total thyroidectomy. However, the clinical significance of these recurrences differed. All 8 patients with regional recurrences after total or near-total thyroidectomy were re-treated by additional surgery, radioiodine ablation, and/or EBRT; of these, 2 achieved complete negative conversion of stimulated serum Tg and two others showed progressive multiple lung metastases with increasing Tg level. Most of these patients had lateral cervical node metastases at initial presentation and underwent modified radical neck dissection and radioiodine treatment, but even these aggressive initial treatments were insufficient to control their remaining disease. These findings suggest that some patients with PTMC have advanced disease with a very aggressive course, despite the small size of the primary tumor, and that these patients should be treated very aggressively, irrespective of primary tumor size.

In the hemi- or subtotal thyroidectomy group, 6 patients had recurrences, most with local tumors in the contralateral lobes without extrathyroid involvement. Five of these patients, who were treated by re-operation and subsequent radioiodine ablation, achieved complete negative conversion of stimulated serum Tg level, indicative of complete remission. These patients may be regarded as having multifocal tumors detected over time, which might show different biological behaviors than the tumors characteristic of persistent disease after initial total or near-total thyroidectomy, with or without radioiodine treatment. Thus, hemi- or subtotal thyroidectomy alone may be sufficient for some PTMC patients without evidence of cervical lymph node metastasis or extrathyroid extension, and clear contralateral lobes, as confirmed by preoperative ultrasonography.

Because PTMC is associated with an excellent prognosis after surgical removal, large numbers of patients with extremely long-term follow-up are needed to identify the clinical relevance of factors prognostic for recurrence [3,4,9-15]. In contrast, we recruited only a relatively small number of eligible PTMC patients, and the median follow-up period was short. The lack of association between previously determined prognostic factors and clinical recurrence in this study may have resulted from these limitations in sample size and follow-up duration. Despite these limitations, our findings suggest the need for larger studies to assess the relationships between lateral neck node metastasis and/or gender with clinical recurrence in patients with PTMC.

The choice of surgical extent for patients with PTMC is unclear, with previous reports showing that 17–91% undergo total or near-total thyroidectomy [3,4,8,9,11-15]. Our results suggest that hemi- or subtotal thyroidectomy may be sufficient for patients with no evidence of cervical lymph node metastasis or extrathyroid extension and clear contralateral lobes. It has been suggested, however, that a significant proportion of patients with PTMC without regional metastasis can be managed by observation alone or by suppression therapy with follow-up using ultrasonography [20,21], but no long-term follow-up studies are available to support this approach. Appropriately extensive surgery, according to prognostic parameters such as lateral cervical node metastasis and gender, may therefore be the standard therapy for PTMC patients.

## Conclusion

PTMCs showed very diverse clinical manifestations, with some having very aggressive clinical behavior, despite small primary tumor size. Clinical recurrence was associated with lateral cervical lymph node metastasis and the male gender, but not with the presence of microscopic extrathyroid extension. Multifocal diseases should be treated with total or near-total thyroidectomy followed by radioactive iodine ablation. In some patients with lateral cervical node involvement, very aggressive treatment is mandatory; but in some patients with unilateral disease limited to one lobe, unilateral thyroidectomy with attentive follow-up may be sufficient. Selection of proper management strategies for individual patients should be based on the results of longer-term follow-up studies with larger numbers of patients.

## Competing interests

The authors declare that they have no competing interests.

## Authors' contributions

TYK drafted the manuscript. SJH carried out operation of the patients and participated in acquisition of informed consent and preparing the manuscript. JMK participated in collection of data and participated in study design. WGK performed the statistical analysis and participated in revision of manuscript. GG reviewed all the pathology slides of the study subject. JSR and WBK participated in acquisition of recurrence data in study subjects and coordinated the study. SCY is a professional statistician and performed the re-analysis of the data in revised manuscript. YKS conceived of the study and participated in the development of manuscript and revision. All authors have read and approved the final manuscript

## Pre-publication history

The pre-publication history for this paper can be accessed here:


